# Patterns, causes, and clinical impact of treatment interruption on early outcomes in patients receiving radiotherapy for lung cancer: a retrospective study

**DOI:** 10.3389/fonc.2026.1894291

**Published:** 2026-08-31

**Authors:** Lifa Su, Longbin Zhang, Qiang Liu, Xin He, Xiaoqing Luo

**Affiliations:** Department of Tumor Center, Chongqing University Three Gorges Hospital, Chongqing, China

**Keywords:** cancer, lung cancer, occurrence pattern, radiotherapy, treatment interruption

## Abstract

**Background:**

Radiation therapy is key for lung cancer, but data on unplanned interruptions remains limited.

**Objectives:**

This study investigated patterns, risk factors, and clinical impacts of radiotherapy interruptions in lung cancer patients.

**Methods:**

A retrospective study enrolled 230 lung cancer patients undergoing radiotherapy (Jan 2020‐Dec 2022). After exclusion, 226 patients were analyzed and stratified into interrupted and non-interrupted groups by unplanned radiotherapy interruptions (≥1-day pause, excluding scheduled breaks). Patterns and causes of interruptions were documented in detail. Early oncological response, treatment completion, acute toxicities, progression-free survival (PFS), and overall survival (OS) were monitored at 6 months post-radiotherapy. Multivariable linear regression identified interruption risk factors, while Cox regression and Kaplan–Meier analysis with log-rank test evaluated survival.

**Results:**

A total of 226 lung cancer patients were enrolled, 79 (34.96%) of whom had at least one treatment interruption, with a median duration of 5 days and 1 episode. Most interruptions occurred in the middle third of radiotherapy (60.76%), mainly caused by treatment-related toxicities (49.37%) and patient-related factors (27.85%). Multivariable linear regression showed that performance status (PS) score 2–3, stage III–IV and tumor volume ≥50 cm³ remained significantly associated with radiotherapy interruption after adjustment for potential confounders (all P<0.05). The interrupted group had poorer compliance, lower overall response rate (ORR) and disease control rate (DCR), higher acute toxicities. Unadjusted Kaplan-Meier analysis showed worse 6-month PFS and OS in the interrupted group, and this association persisted in multivariable-adjusted Cox regression after controlling for PS score, tumor stage, tumor volume, treatment regimen, and radiotherapy modality (all P < 0.001).

**Conclusions:**

Radiotherapy interruption is common in lung cancer patients, mainly caused by toxicity. It impairs treatment efficacy and survival, requiring individualized management.

## 1 Introduction

Lung cancer remains one of the most prevalent and lethal malignancies worldwide, ranking first in both incidence and mortality among all cancers in China (1, 2). Radiotherapy is a cornerstone of lung cancer management across all stages—from radical treatment for inoperable early-stage disease to combined therapy for locally advanced cases and palliative care for metastatic disease (3, 4).

The introduction of stereotactic body radiation therapy (SBRT) has provided long-term survival rates comparable to surgical resection for patients with inoperable early-stage lung cancer, with 5-year local control rates exceeding 85% to 95%, establishing it as a definitive treatment option in this population (5). In the setting of unresectable locally advanced non-small cell lung cancer (NSCLC), concurrent chemoradiotherapy remains the widely accepted gold standard, and its combination with immunotherapy consolidation has been shown to further improve overall survival (6, 7). In patients with advanced disease, palliative radiotherapy effectively alleviates symptoms such as pain and intracranial hypertension caused by bone and brain metastases (8, 9). In recent years, the widespread adoption of precision radiotherapy techniques — including image-guided radiation therapy, respiratory gating, and artificial intelligence-assisted target delineation and plan optimization — has further improved treatment efficacy and helped control treatment-related adverse events to some extent. Despite these therapeutic advances, unplanned interruptions of radiotherapy continue to occur frequently in clinical practice and have become a major barrier to achieving optimal therapeutic outcomes (10).

The effectiveness of radiotherapy depends on the continuous delivery of ionizing radiation to tumor cells. Treatment outcomes are closely tied to both the complete delivery of the prescribed dose and the continuity of the treatment course. Any unplanned interruption may permit tumor cells to repair sublethal damage and undergo accelerated repopulation, thereby reducing local control and increasing the risks of recurrence and distant metastasis (11). Previous studies have suggested that interruptions exceeding five days significantly compromise efficacy, whereas shorter pauses of one to two days may have a relatively minor impact, indicating a dose-response relationship between interruption duration and clinical outcomes (12).

Current evidence on radiotherapy interruptions in lung cancer remains limited in several key aspects. First, reported interruption rates vary widely across studies (ranging from 20% to 45%) (13), likely due to heterogeneity in study populations, treatment regimens, and institutional practices. Second, most existing studies focus on a single cause of interruption or only preliminarily explore its association with one clinical endpoint, which limits their ability to inform targeted interventions (14). Third, updated guidelines (15) have expanded the use of combined modalities such as concurrent chemoradiotherapy and radio-immunotherapy, which may increase toxicity and further challenge treatment continuity—yet the risk factors for interruption in this evolving treatment landscape have not been systematically reassessed. Fourth, the relationships between clinically relevant factors (PS score, tumor stage, tumor volume, treatment regimen) and radiotherapy interruption remain inconsistent across studies, and the underlying mechanisms are poorly understood (16).

Early clinical outcomes—including tumor response, treatment completion rates, acute toxicities, and short-term survival—are critical indicators for evaluating treatment efficacy and predicting long-term prognosis. Radiotherapy interruptions may reduce the actual delivered dose, lower treatment completion rates, and thereby impair early oncological efficacy (17). Interruptions may also allow tumor burden to increase, worsen clinical conditions, and ultimately lead to inferior short-term survival. However, the precise magnitude and pathways through which radiotherapy interruptions affect these early endpoints remain unclear, making it difficult to establish a definitive causal relationship (18).

In the era of personalized and precision medicine, improving patient prognosis and quality of life is the central goal of clinical practice. Reducing unplanned interruptions and maintaining treatment continuity are essential steps toward optimizing radiotherapy outcomes (19). Nevertheless, a systematic investigation that simultaneously addresses the patterns, detailed causes, independent risk factors, and early clinical impacts of radiotherapy interruptions in a unified cohort is still lacking.

In response to this gap, the present study employed a retrospective cohort design. We comprehensively analyzed the patterns and etiologies of radiotherapy interruptions, identified independent risk factors, and evaluated their impacts on early oncological response, treatment completion, acute toxicities, and short-term survival. Our findings aim to provide reliable clinical evidence for developing targeted interventions, streamlining treatment workflows, implementing personalized management, minimizing unplanned radiotherapy interruptions.

## 2 Patients and methods

### 2.1 Study design

**Figure 1:**
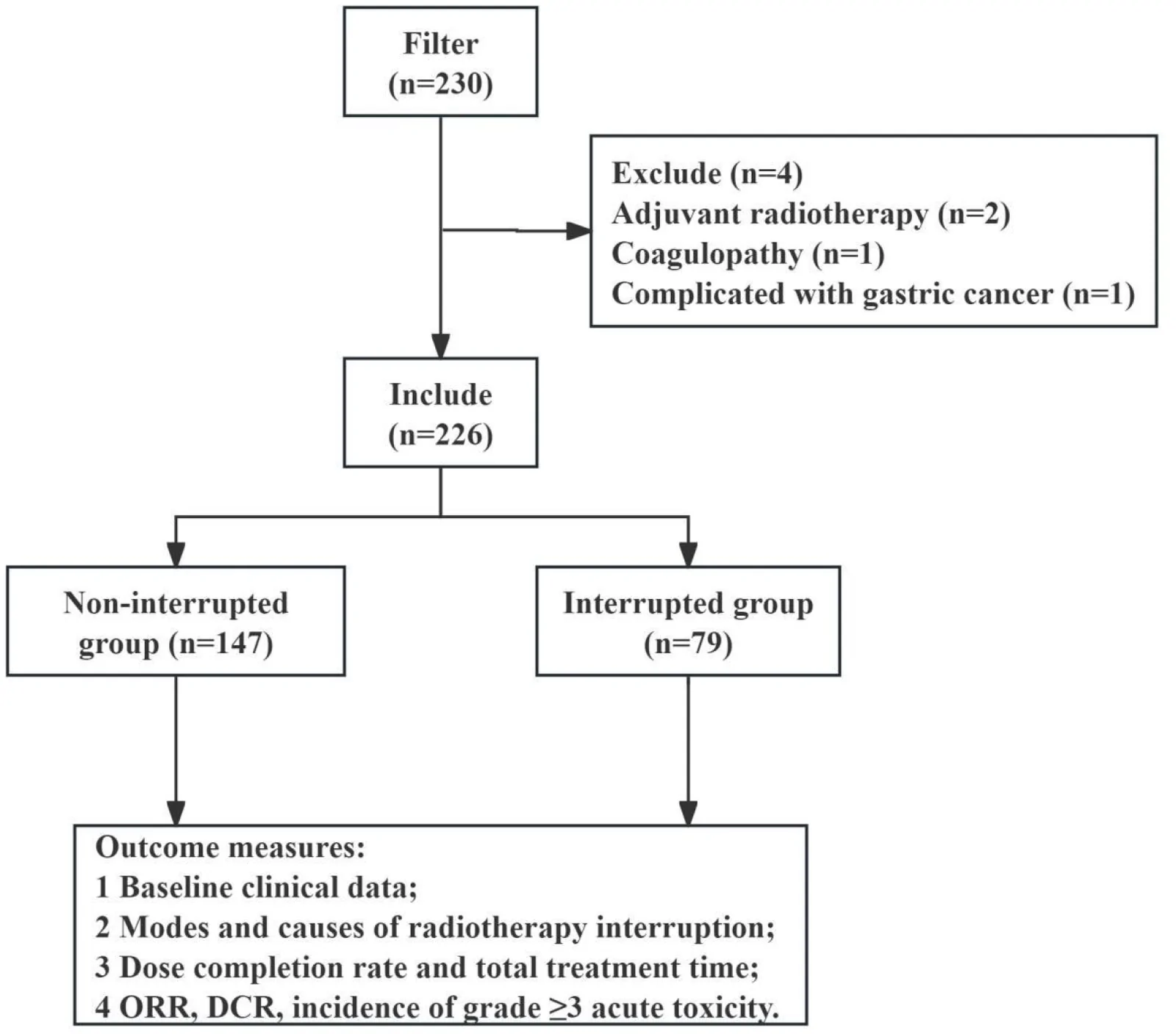
Research Flowchart. 230 patients were initially screened in this study, after exclusion, 226 cases were included, with 147 cases in non-interrupted group and 79 cases in interrupted group. ORR, Overall Response Rate; DCR, Disease Control Rate.

230 lung cancer patients who received either radical or palliative radiotherapy at Chongqing University Three Gorges Hospital (Jan 2020 and Dec 2022) were initially enrolled in this study. Subsequent to the application of exclusion criteria, 226 cases were ultimately included for formal analysis. These patients were then divided into the interrupted group and the non-interrupted group. Unplanned interruptions were defined as consecutive treatment pauses of ≥1 day, excluding scheduled breaks built into the radiotherapy protocol, This cutoff was selected to provide a maximally sensitive definition for this exploratory study, aiming to capture all potential interruption events (**Figure 1**).

### 2.2 Inclusion criteria

(1) Patients with a histologically or cytologically confirmed diagnosis of lung cancer, including NSCLC and small cell lung cancer (SCLC), in line with the diagnostic criteria specified in the Guidelines for the Clinical Diagnosis and Treatment of Lung Cancer (2024 Edition) issued by the Chinese Medical Association (15); (2) Age ranging from 18 to 80 years old, with no restrictions on gender; (3) Undergoing radical or palliative radiotherapy at our hospital, with a clearly defined radiotherapy regimen, a planned total radiation dose of at least 50 Gy, and a planned treatment fraction number of 20 or more; (4) Availability of complete and valid clinical data; (5) PS score of 3 or lower, with no severe dysfunction of the heart, liver, kidneys, hematopoietic system, or other vital organs, and the ability to tolerate radiotherapy and related examinations; (6) Feasibility of complete follow-up for at least 6 months after radiotherapy, with reliable documentation of early clinical outcomes and survival status.

### 2.3 Exclusion criteria

(1) Patients with an unclear pathological diagnosis, uncertain histological type or tumor stage, or those with a history of other malignant tumors; (2) Patients receiving prophylactic or adjuvant radiotherapy, or those with an ill-defined treatment plan, a planned radiation dose of less than 50 Gy, or fewer than 20 treatment fractions; (3) Patients whose treatment was interrupted due to scheduled rest periods or transfer to another hospital, rather than unplanned radiotherapy interruptions; (4) Patients with severe psychiatric disorders, coagulopathy, active infections unrelated to lung cancer, or other severe comorbidities that could significantly interfere with treatment tolerance, study compliance, or accurate outcome assessment.

### 2.4 Radiotherapy regimens

In this study, personalized radiotherapy strategies were designed for all enrolled patients in accordance with the Guidelines for the Clinical Diagnosis and Treatment of Lung Cancer (2024 Edition, 2024) (15). Treatment decisions were determined based on tumor stage, pathological classification, PS, and therapeutic purpose, with patients receiving either radical or palliative radiotherapy. All procedures were performed using three-dimensional conformal radiotherapy (3D-CRT) or intensity-modulated radiotherapy (IMRT) techniques. Experienced radiation oncologists precisely delineated target volumes, based on imaging findings.

Radical radiotherapy was mainly used for patients with early-stage inoperable or locally advanced unresectable lung cancer. The planned dose spanned 60 to 70 Gy, given in 30 to 35 fractions, once daily, five days per week. To improve treatment efficacy, some patients underwent concurrent chemotherapy or immunotherapy with programmed cell death protein 1/programmed death-ligand 1 (PD-1/PD-L1) inhibitors (20). The chemotherapeutic agents included Pemetrexed Disodium for Injection (100 mg per vial, boxed; Shandong New Time Pharmaceutical Co., Ltd., Shandong, China);Cisplatin Injection (10 mg:2 mL per vial; Yunnan Phytopharmaceutical Co., Ltd., Yunnan, China);Paclitaxel Injection (5 mL:30 mg per vial, boxed; Yangtze River Pharmaceutical Group Co., Ltd., Jiangsu, China; or 100 mg per vial, boxed; Qilu Pharmaceutical (Hainan) Co., Ltd., Hainan, China);Carboplatin Injection (100 mg:10 mL per ampoule; Qilu Pharmaceutical Co., Ltd., Shandong, China). The immunotherapeutic agents used were Tirelizumab Injection (10 mL:100 mg per vial; Guangzhou BeiGene Biopharmaceutical Co., Ltd., Guangdong, China);Serplulimab Injection (100 mg:10 mL per vial, boxed; Shanghai Henlius Biopharmaceutical Co., Ltd., Shanghai, China);Bevacizumab Injection (100 mg (4 mL) per vial, boxed; Innovent Biologics (Suzhou) Co., Ltd., Jiangsu, China; or 100 mg (4 mL) per vial, boxed; Nanjing Shunxin Pharmaceutical Co., Ltd., Chia Tai Tianqing Pharmaceutical Group, Nanjing, China). The targeted therapeutic agent used was Furmonertinib Mesylate (40 mg × 28 tablets per box; Jiangsu Allist Pharmaceuticals Co., Ltd., Jiangsu, China).

Palliative radiotherapy was administered to patients with advanced lung cancer and distant metastases, including bone, brain, and mediastinal lymph node metastases. The prescribed dose was 30 to 50 Gy in 10 to 25 fractions, once daily, five days per week. The primary aims were to alleviate symptoms caused by metastatic lesions, control tumor progression, and improve short-term quality of life (21). When combined systemic therapy was needed during palliative radiotherapy, the same medications used in the radical radiotherapy group were applied.

### 2.5 Data extraction

Two investigators who had received systematic and standardized training independently reviewed clinical materials of the included subjects, such as electronic medical records and radiotherapy treatment documents. The entire data extraction procedure was carried out under the double-checking and cross-verification principle. Any inconsistency or discrepancy identified during data collection was further evaluated and determined by a third senior researcher to ensure accuracy and reliability. Information extracted in this study mainly covered demographic characteristics, baseline tumor features, and treatment-related parameters. In addition, detailed data concerning radiotherapy interruptions were documented separately. Treatment-related adverse events were graded and recorded in accordance with the CTCAE 5.0 criteria, together with early clinical outcomes.

### 2.6 Outcome measures

#### 2.6.1 Outcomes related to radiotherapy interruption

Unplanned radiotherapy interruption was defined as a continuous treatment pause of ≥1 day during the course of radiotherapy, excluding scheduled breaks such as regular weekly days off and predefined intervals. The timing of interruption (early one-third, middle one-third, or late one-third of treatment), duration (consecutive days from the start of interruption to resumption), frequency, and specific causes were all documented (22). Causes of interruption were determined comprehensively based on patient symptoms, laboratory findings, nursing records, and physician-patient communications. Interruptions caused by treatment-related toxicities were confirmed and graded as described in the CTCAE 5.0 system (23).

#### 2.6.2 Outcomes related to treatment completion

Indicators included the relative dose completion rate (ratio of the actual delivered dose to the prescribed dose) and the number of days prolonging overall treatment duration (difference between actual and planned total treatment days) (24). A dose completion rate ≥90% was defined as adequate dose fulfillment, while a rate <90% was regarded as insufficient (25). Planned and actual radiotherapy doses, as well as planned and actual treatment durations, were extracted from treatment records, and the above indices were calculated using standard formulas.

#### 2.6.3 Early oncologic efficacy outcomes

Overall response rate (ORR) and disease control rate (DCR) were evaluated in accordance with the Response Evaluation Criteria in Solid Tumors (RECIST, version 1.1). Complete response (CR) was defined as the complete disappearance of all target lesions for at least 4 weeks. Partial response (PR) was defined as a ≥30% reduction in the sum of the longest diameters of target lesions from baseline for at least 4 weeks. Stable disease (SD) was defined as a <30% decrease or <20% increase in the sum of the longest diameters. Progressive disease (PD) was defined as a ≥20% increase in the sum of the longest diameters or the appearance of new lesions. ORR = (CR + PR)/total number of patients × 100%. DCR = (CR + PR + SD)/total number of patients × 100% (26).

#### 2.6.4 Acute toxicity outcomes

Treatment-related adverse events occurring during radiotherapy and within 6 months after treatment were recorded, mainly including radiation esophagitis, radiation pneumonitis, myelosuppression, and radiation dermatitis. These events were graded from 0 to 5 using CTCAE 5.0, and the present study was centered around the incidence of acute toxicities of grade 3 or higher (23). During radiotherapy, patients were monitored weekly for symptoms and physical signs. Routine blood tests (white blood cells, platelets, hemoglobin) were performed using a BC-6800 analyzer (Sysmex XN20, Sysmex Corporation, Japan).Diagnostic assessments also included chest computed tomography (Revolution CT, General Electric Company, USA) for pulmonary inflammation and, when necessary, esophagoscopy (GIF-H290Z, Olympus Medical Systems Corp., Japan).

#### 2.6.5 Short-term survival outcomes

Progression-free survival (PFS) and overall survival (OS) were assessed. PFS was defined as the interval from the initiation of radiotherapy to disease progression or death from any cause. OS was defined as the interval from the start of radiotherapy to death from any cause or the follow-up time point of 6 months after radiotherapy (27).

### 2.7 Ethical statement

This study was performed in strict accordance with the principles outlined in the Declaration of Helsinki. All study procedures were reviewed and approved by the Ethics Committee. Written informed consent was voluntarily obtained from all participants prior to study enrollment.

### 2.8 Sample size calculation

The sample size estimation was performed for the primary between-group comparison, i.e., testing the difference in a key binary clinical outcome between the interrupted and non-interrupted groups, using a two-independent-proportions test. According to a previous investigation (28), approximately 18% of patients with NSCLC undergoing radiotherapy experienced unplanned treatment interruptions. Assuming a clinically meaningful difference between the two groups in the primary efficacy endpoint (e.g., ORR of ~55% in the interrupted group vs. ~75% in the non-interrupted group), the resulting standardized effect size (Cohen’s h) was 0.36, as calculated using G*Power 3.1.9.7 software. A two-sided test was adopted, with a type I error rate (α) set at 0.05 and a type II error rate (β) set at 0.05. Based on these parameters, the minimum required sample size was estimated to be 172 individuals. It should be noted that this power calculation primarily applies to the above group comparison, rather than to the multivariable Cox regression analysis-for the latter, given the event-per-variable (EPV) rule (typically requiring 10–15 events per covariate), the actual number of events in our study met this empirical requirement, and no formal power estimation was performed. In the present study, a final total of 226 patients were included, which exceeded the calculated threshold. *Post hoc* power analysis was performed on the 226 included patients using G*Power 3.1.9.7. Results demonstrated that, at a significance level of α = 0.05, statistical power for the primary endpoints was no less than 80%. This finding further confirmed that the sample size employed in this research was rational, reliable, and met the statistical requirements for a retrospective study.

### 2.9 Statistical analysis

Logistic regression was applied to adjust for potential confounding biases derived from baseline characteristics. For continuous variables, normality was assessed using the Shapiro-Wilk test, and homogeneity of variance was examined via the Levene test. Variables that followed a normal distribution and exhibited equal variance were presented as the mean ± standard deviation (mean ± SD). Between-group comparisons were performed using independent-samples t-tests, whereas paired t-tests were used for within-group comparisons. Variables that failed to satisfy normality assumptions were expressed as the median (interquartile range) [M (Q1, Q3)].Between-group differences were analyzed using the Mann-Whitney U test, and within-group differences were evaluated with the Wilcoxon signed-rank test. Categorical data were summarized as counts and percentages [n (%)], and intergroup comparisons were carried out using the chi-square test. Variables with a P-value < 0.10 in univariate analyses were introduced into a multivariable linear regression model to adjust for potential confounders. This analysis aimed to explore the independent associations of various factors with radiotherapy interruption, rather than to build a clinical prediction model for individual risk prediction. The threshold of P<0.10 was used as an exploratory screening criterion, and the results should be interpreted in conjunction with clinical plausibility and validated in future studies. Survival curves for the interrupted and non-interrupted groups were constructed using the Kaplan-Meier method, and differences were compared using the log-rank test to evaluate the impact of radiotherapy interruption on short-term survival outcomes. Multivariable Cox proportional hazards regression analysis was further conducted, incorporating radiotherapy interruption along with the following prespecified potential confounders, which were selected based on both clinical knowledge (established prognostic factors for lung cancer) and univariate screening (P<0.10): PS score (0–1 vs. 2–3), tumor stage (I–II vs. III–IV), tumor volume (<50 vs. ≥50 cm³), treatment regimen (non-concurrent vs. concurrent chemoradiotherapy), and radiotherapy modality (definitive vs. palliative). This approach was used to verify whether radiotherapy interruption served as an independent adverse prognostic factor for short-term PFS and OS, while simultaneously controlling for confounding effects. All statistical tests were two-sided, and P < 0.05 was considered statistically significant.

## 3 Results

### 3.1 Baseline characteristics

Among 226 patients, 79 patients experienced treatment interruption, corresponding to an overall interruption rate of 35.0%. Accordingly, the study population was divided into an interrupted group (n = 79) and a non-interrupted group (n = 147). Baseline clinical characteristics were then compared between the two groups. No statistically significant differences were observed in terms of age, sex, body mass index (BMI), smoking history, comorbidities, or pathological type (all P > 0.05). These findings indicated that the two groups were well balanced with respect to basic demographic and clinical features, and thus were comparable. In contrast, significant between-group differences were detected in five key indicators: tumor stage, tumor volume, PS score, radiotherapy modality, and overall treatment strategy (all P < 0.05) (**Table 1**). Patients with more advanced tumor stage, larger tumor volume, poorer functional status, palliative radiotherapy, or a higher proportion of concurrent chemoradiotherapy were more prone to developing radiotherapy interruptions. These baseline differences provide important contextual information for the subsequent evaluation of how treatment interruption affects therapeutic efficacy and clinical prognosis.

**Table 1:** Baseline clinical data.

Indicators	Non-interrupted group (n=147)	Interrupted group (n=79)	*P*	OR	95%CI for OR
Age (years, mean ± SD)	61.25 ± 1.80	61.84 ± 4.68	0.186	1.07	0.97,1.18
Gender (n %)			0.818	0.94	0.54,1.64
Male	87 (59.2)	48 (60.8)			
Female	60 (40.8)	31 (39.2)			
BMI (kg/m^2^, mean ± SD)	22.36 ± 1.27	22.56 ± 0.74	0.185	1.18	0.93,1.50
Smoking history (n %)			0.511	0.83	0.48,1.44
Yes	64 (43.5)	38 (48.1)			
No	83 (56.5)	41 (51.9)			
History of comorbidities (n %)			0.274	0.73	0.41,1.29
Yes (COPD/CHD, etc.)	47 (32.0)	31 (39.2)			
No	100 (68.0)	48 (60.8)			
Pathological type (n %)			0.947	1.03	0.47,2.27
NSCLC	127 (86.4)	68 (86.1)			
SCLC	20 (13.6)	11 (13.9)			
Tumor stage (AJCC, n %)			<0.001	3.38	1.65,6.96
I‐II	52 (35.4)	11 (13.9)			
III‐IV	95 (64.6)	68 (86.1)			
Tumor volume (cm^3^, mean ± SD)	41.49 ± 3.30	59.09 ± 3.34	<0.01	1.97	1.48,2.64
PS score (ECOG, n %)			<0.001	4.88	2.70,8.81
0‐1	113 (76.9)	32 (40.5)			
2‐3	34 (23.1)	47 (59.5)			
Radiotherapy type (n %)			0.002	0.41	0.23,0.72
Definitive radiotherapy	97 (66.0)	35 (44.3)			
Palliative radiotherapy	50 (34.0)	44 (55.7)			
Treatment regimen (n %)			0.044	1.48	1.01,2.16
Radiotherapy alone	71 (48.3)	21 (26.6)			
Concurrent chemoradiotherapy	50 (34.0)	45 (57.0)			
Radiotherapy combined with immunotherapy	26 (17.7)	13 (16.4)			

OR, Odds Ratio; 95%CI, 95% Confidence Interval; BMI, Body Mass Index; COPD, Chronic Obstructive Pulmonary Disease; CHD, Coronary Heart Disease; NSCLC, Non-Small Cell Lung Cancer; SCLC, Small Cell Lung Cancer; AJCC, American Joint Committee on Cancer; PS, Performance Status; ECOG, Eastern Cooperative Oncology Group.

### 3.2 Patterns and causes of treatment interruption

Detailed characteristics related to radiotherapy interruptions are systematically summarized in **Table 2**. The median duration of interruption was 5 days, and most patients experienced only a single interruption, with a median number of 1 episode. The timing of interruptions was unevenly distributed across the treatment course, with most events occurring during the middle phase. Specifically, 17 patients (21.5%) developed an interruption in the first third of radiotherapy, 48 patients (60.8%) in the middle third, and 14 patients (17.7%) in the final third, revealing a markedly higher incidence during the mid-treatment period. Causes of interruption were categorized into five major groups. Treatment-related toxicities represented the leading cause, accounting for 39 cases (49.4%). These mainly consisted of grade ≥3 radiation esophagitis in 18 patients (22.8%), grade ≥3 radiation pneumonitis in 11 patients (13.9%), grade ≥3 myelosuppression in 7 patients (8.9%), and grade ≥2 radiation dermatitis in 3 patients (3.8%). Patient-related factors were the second most common cause, documented in 22 cases (27.8%).This subgroup included family issues in 8 patients (10.1%), transportation difficulties in 6 patients (7.6%), financial constraints in 5 patients (6.3%), and fear of treatment in 3 patients (3.8%). Acute exacerbations of other systemic diseases accounted for 10.1% (8 cases), mainly acute exacerbation of chronic obstructive pulmonary disease in 4 patients (5.1%), acute pulmonary infection in 3 patients (3.8%), and acute coronary events in 1 patient (1.3%). Disease progression was responsible for 6 interruptions (7.6%), all of which were attributed to local tumor progression or distant metastasis. Institutional factors were the least frequent cause, noted in 4 cases (5.1%), including equipment malfunction and temporary schedule adjustments due to holidays, with 2 cases (2.5%) for each. Overall, radiotherapy interruptions in lung cancer patients were predominantly single, short-duration events occurring in the middle of treatment. Treatment-related toxicity was identified as the primary cause, while patient characteristics and pre-existing comorbidities also contributed substantially. These results may offer valuable clues for the design of targeted interventions in future clinical practice.

**Table 2:** Patterns and causes of radiotherapy interruption.

Variable/category	Value/subcategory, n (%) or M (Q1, Q3)
Duration of interruption (days)	5 (4, 7)
Number of interruptions (times)	1 (1, 2)
Stage of interruption occurrence (n=79)	
- First one-third stage before radiotherapy	17 (21.5)
- Middle one-third stage during radiotherapy	48 (60.8)
- Last one-third stage during radiotherapy	14 (17.7)
Causes of interruption (n=79)	
Treatment-related toxic reactions	39 (49.4)
- Grade ≥3 radiation esophagitis	18 (22.8)
- Grade ≥3 radiation pneumonitis	11 (13.9)
- Grade ≥3 myelosuppression	7 (8.9)
- Grade ≥2 radiation dermatitis	3 (3.8)
Patient-related factors	22 (27.8)
- Family affairs	8 (10.1)
- Inconvenient transportation	6 (7.6)
- Financial hardship	5 (6.3)
- Fear of treatment	3 (3.8)
Acute exacerbation of other systemic diseases	8 (10.1)
- Acute exacerbation of chronic obstructive pulmonary disease	4 (5.1)
- Acute pulmonary infection	3 (3.8)
- Acute CHD attack	1 (1.3)
Disease progression	6 (7.6)
- Local tumor progression/distant metastasis	6 (7.6)
Institutional factors	4 (5.1)
- Equipment failure	2 (2.5)
- Temporary holiday adjustment	2 (2.5)

### 3.3 Factors independently associated with radiotherapy interruption in multivariable linear regression analysis

This study adopted multivariable linear regression to adjust for confounders, aiming to explore the independent associations between each clinical indicator and the number of radiotherapy interruption days, rather than constructing a predictive model for individual risk assessment (**Table 3**). Among the five covariates investigated, tumor stage (III–IV vs. I–II; β = 0.239, P = 0.038, B = 0.827, 95% CI: 0.046–1.608), tumor volume (≥50 vs. <50 cm³; β = 0.231, P = 0.047, B = 1.675, 95% CI: 0.026–3.325), and PS score (2–3 vs. 0–1; β = 0.277, P = 0.044, B = 0.884, 95% CI: 0.024–1.743) achieved statistical significance, suggesting that these three variables serve as independent correlates of radiotherapy interruption. Conversely, treatment regimen (concurrent chemoradiotherapy vs. non-concurrent; β = −0.092, P = 0.413, B = −0.295, 95% CI: −1.009 to 0.419) and radiotherapy type (palliative vs. definitive; β = −0.100, P = 0.483, B = −0.325, 95% CI: −1.243 to 0.594) did not show significant associations, indicating that neither factor was independently associated with interruption days.

**Table 3:** Multivariable linear regression analysis of factors associated with radiotherapy interruption.

Indicators	*β*	*P*	B	95% for B
Tumor stage (I‐II/III‐IV)	0.239	0.038	0.827	0.046, 1.608
Tumor volume (<50cm^3^/≥50cm^3^)	0.231	0.047	1.675	0.026, 3.325
PS score (0-1/2-3)	0.277	0.044	0.884	0.024, 1.743
Treatment regimen (non-concurrent chemoradiotherapy/concurrent chemoradiotherapy)	-0.092	0.413	-0.295	-1.009, 0.419
Radiotherapy type (definitive radiotherapy/palliative radiotherapy)	-0.100	0.483	-0.325	-1.243, 0.594

### 3.4 Dose completion rate and total treatment duration

See **Table 4**, the dose completion rate in the non-interrupted group was significantly higher than that in the interrupted group (P < 0.001).When it comes to total treatment duration, the non-interrupted group also showed a marked advantage—it was notably shorter than the interrupted group (P < 0.001). Taken together, these findings suggest an obvious association, radiotherapy interruption is linked to both a reduced dose completion rate and a prolonged total treatment duration in patients.

**Table 4:** Comparison of dose completion rate and total treatment time.

Indicators	Non-interrupted group (n=147)	Interrupted group (n=79)	*P*	95%CI of the difference	Effect size (Cohen’s D)
Dose completion rate (%, mean ± SD)	95.04 ± 1.17	81.89 ± 2.49	<0.001	12.56,13.73	7.529
Total treatment time (days, mean ± SD)	1.19 ± 0.48	7.45 ± 1.48	<0.001	-6.60,-5.92	6.559

### 3.5 Early oncologic efficacy indicators

See **Table 5** for detailed data. The ORR in the non-interrupted group reached 76.2%—a figure significantly higher than that in the interrupted group, which stood at 55.7%. This between-group difference was statistically significant (P = 0.001). Similarly, the DCR of the non-interrupted group was obviously higher than that of the interrupted group, 93.9% versus 77.2%, and the difference was of statistical significance (P < 0.001). When it comes to the incidence of grade ≥3 acute toxicities, the non-interrupted group had a notably lower rate—only 19.7%. In sharp contrast, the interrupted group saw a much higher incidence, reaching 44.3% (35/79), and this gap was statistically significant (P < 0.001). These observational data suggest an association between interruption status and poorer early tumor response as well as greater toxicity burden. Further multivariable-adjusted analyses are warranted to validate these associations.

**Table 5:** Comparison of early oncological efficacy indices.

Indicators	Non-interrupted group (n=147)	Interrupted group (n=79)	*P*	Effect size (Phi)
ORR (n %)	112 (76.2)	44 (55.7)	0.001	0.211
DCR (n %)	138 (93.9)	61 (77.2)	<0.001	0.245
Incidence of grade ≥3 acute toxicity (n %)	29 (19.7)	35 (44.3)	<0.001	0.260

ORR, Overall Response Rate; DCR, Disease Control Rate.

### 3.6 6-month PFS

**Figure 2:**
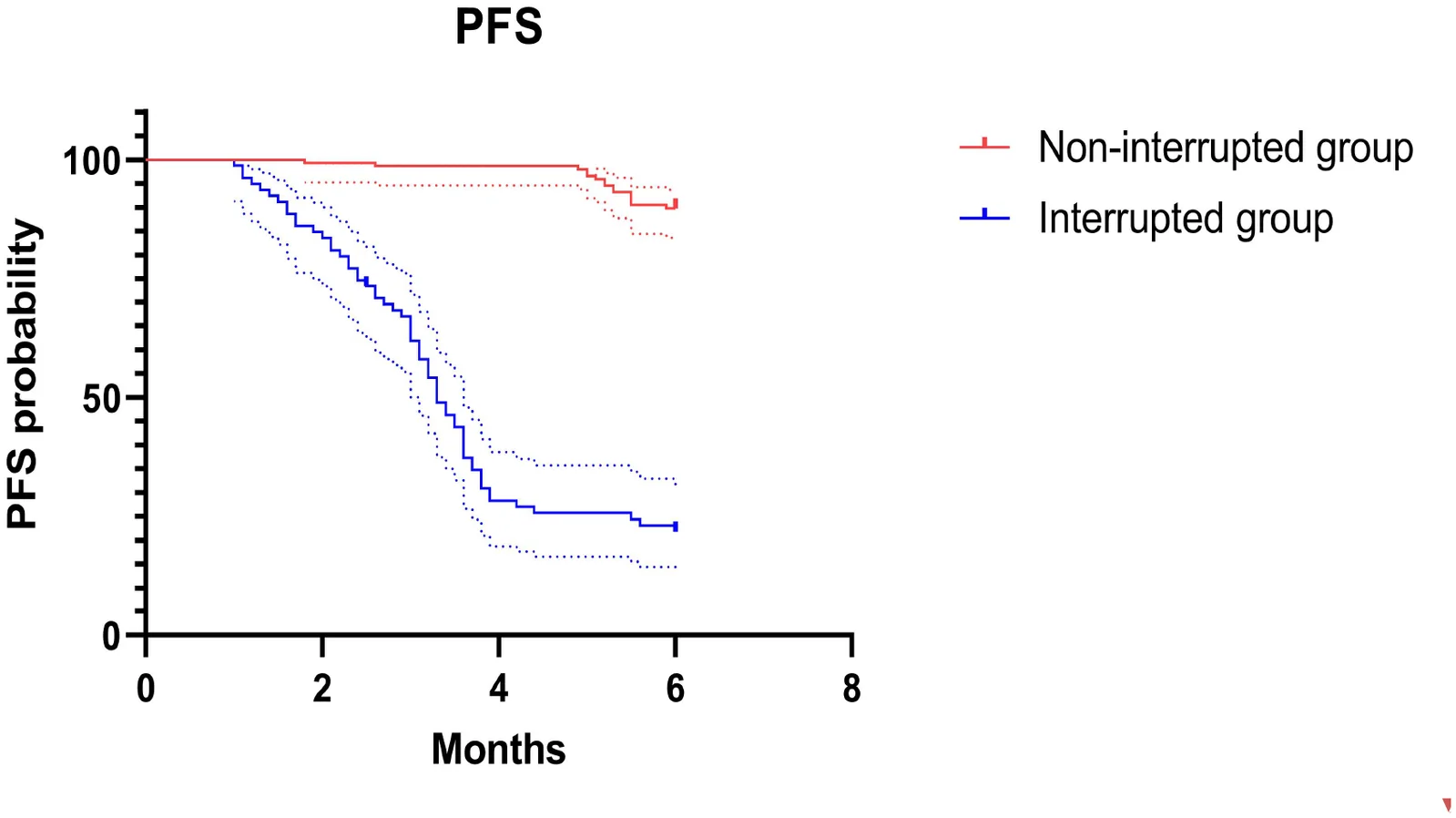
Kaplan-Meier plot of 6-month PFS in the two groups. This image presents the Kaplan-Meier survival curves for 6-month PFS between the two patient groups. PFS, Progression-Free Survival.

Kaplan-Meier survival curves, as illustrated in **Figure 2**, revealed notable differences in PFS between the two groups. The 6-month PFS rate was 89.8% in the non-interrupted group, in stark contrast to only 22.8% in the interrupted group. The non-interrupted group did not reach the median PFS, a finding that may be attributed to the relatively short follow-up duration. In comparison, the interrupted group had a median PFS of 3.3 months (95%CI: 2.99–3.61). Statistical analysis via the Log-rank test showed a statistically significant difference in unadjusted PFS between the two groups (P < 0.001).

### 3.7 6-Month OS

**Figure 3:**
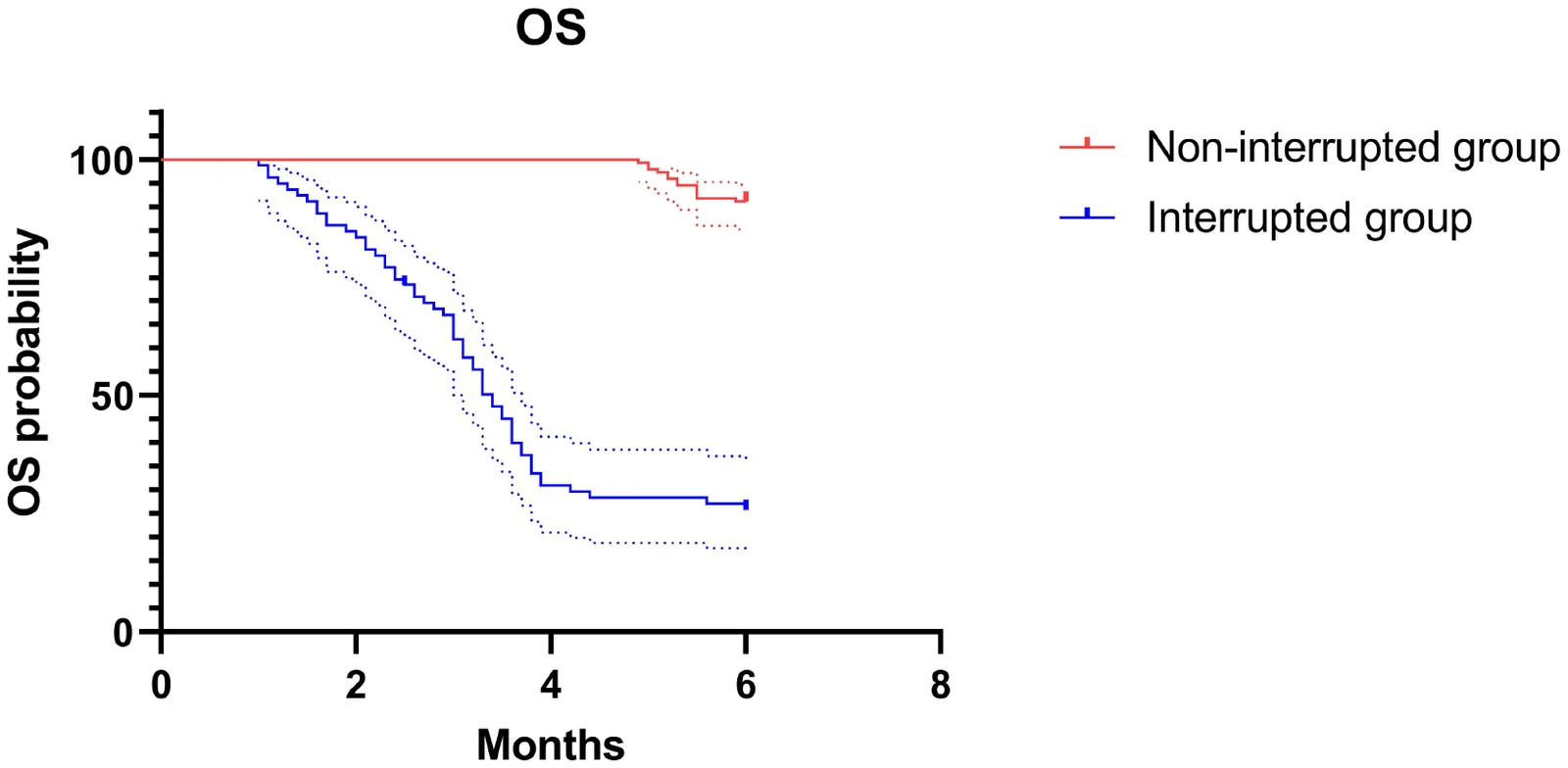
Kaplan-Meier plot of 6-month OS in the two groups. This image displays the Kaplan-Meier survival curves for 6-month OS among the two patient groups. OS, Overall Survival.

See **Figure 3**. The 6-month OS rate stood at 91.2% in the non-interrupted group, whereas it was only 26.6% in the interrupted group. No median OS was achieved in the non-interrupted group. By contrast, the interrupted group had a median OS of 3.4 months (95%CI: 3.11–3.69). The Log-rank test demonstrated a significant difference in unadjusted OS between the two groups (P < 0.001).

### 3.8 Adverse prognostic factors affecting PFS and OS identified by cox regression analysis

**Figure 4:**
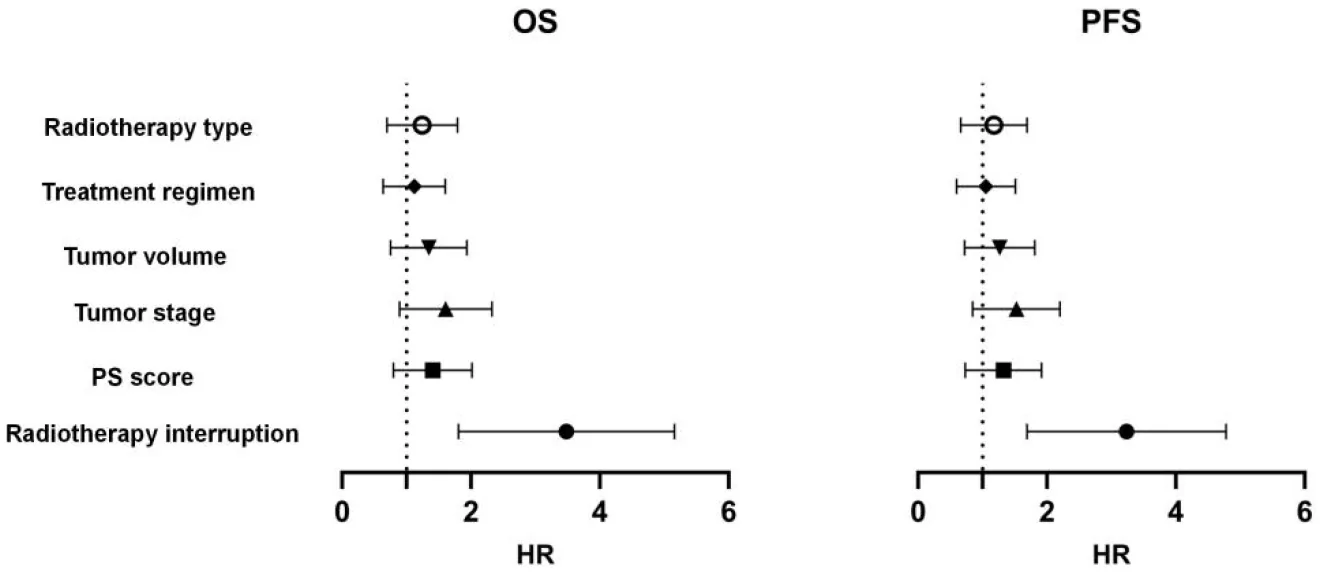
Forest plot of adverse prognostic factors affecting PFS and OS. This image presents a forest plot illustrating the adverse prognostic factors affecting PFS and OS, analyzed by Cox proportional hazards regression. HR, Hazard Ratio.

As shown in **Figure 4**, after adjusting for prespecified potential confounders including PS score, tumor stage, tumor volume, concurrent chemoradiotherapy, and radiotherapy modality (selected based on clinical plausibility and univariate screening P < 0.10), radiotherapy interruption remained associated with both shorter PFS and shorter OS (P < 0.001). Specifically, the adjusted hazard ratio for PFS was 2.987 (95%CI: 1.823-4.895), and for OS was 3.215 (95%CI: 1.956-5.278), indicating that patients with interruption had approximately three-fold higher risks of progression and death than those without interruption after accounting for the above covariates. No statistically significant associations were observed for PS score, tumor stage, tumor volume, concurrent chemoradiotherapy, or radiotherapy modality in this model (all P > 0.05).

## 4 Discussion

In this study, with a total of 226 lung cancer patients who received radiotherapy enrolled for analysis. According to the findings, the incidence of radiotherapy interruption among these patients was 34.96%. The median duration of interruption was 5 days, and the median number of interruptions was 1. Notably, treatment interruptions showed clear phase-specific distribution, with more than 60% of events occurring during the middle one-third of radiotherapy, accounting for 60.76% of all interruptions. Treatment-related toxicity represented the leading cause, responsible for 49.37% of all interruptions, among which grade ≥3 radiation esophagitis, radiation pneumonitis, and myelosuppression were most frequently observed. Patient-related factors followed, contributing to 27.85% of cases, including family issues, transportation difficulties, financial constraints, and fear of treatment. Multivariable linear regression analysis showed that, after adjusting for potential confounders, a PS score of 2-3, advanced tumor stage (III-IV), and a tumor volume ≥50 cm (3) remained significantly associated with radiotherapy interruption in this cohort. In terms of treatment execution, the interrupted group had lower dose completion and longer overall treatment duration than the non-interrupted group, along with lower ORR and DCR and a higher observed incidence of grade ≥3 acute toxicities. Given that toxicity was the predominant cause of interruption, the latter association is more plausibly explained by higher baseline toxicity burden in the interrupted group rather than a causal effect of interruption on adverse events. Kaplan-Meier analysis (unadjusted) demonstrated significantly shorter PFS and OS in the interrupted group. This association was further evaluated in multivariable-adjusted Cox regression, which confirmed that radiotherapy interruption remained significantly associated with worse PFS and OS after controlling for potential confounders. These results suggest that radiotherapy interruption is closely associated with early treatment response and short-term survival prognosis in patients with lung cancer.

In the present study, most radiotherapy interruptions were detected during the middle one-third of the treatment course, which may be attributed to the cumulative effect of radiation dose and the temporal pattern of normal tissue response. During the initial one-third of radiotherapy, patients generally maintain relatively good tolerance because radiation-induced tissue damage remains minimal. As treatment proceeds, however, accumulated radiation exposure gradually triggers overt normal tissue injury, and treatment-related toxicity peaks. Many patients are unable to tolerate severe symptoms and thus require treatment suspension, explaining why the middle phase carries the highest interruption risk. In the final one-third of treatment, patients tend to adapt to persistent but manageable toxicities. As treatment nears completion, adherence often improves, and clinicians can implement targeted interventions for earlier toxicities, leading to a lower interruption rate (29). The predominance of treatment-related toxicity may be linked to the anatomical location of lung cancer and the characteristics of thoracic radiotherapy. The lung is highly radiosensitive, and high-energy rays that target malignant cells inevitably damage adjacent normal lung parenchyma, esophageal mucosa, and bone marrow hematopoietic stem cells, thereby inducing radiation pneumonitis, esophagitis, and myelosuppression (30, 31). Among these toxicities, radiation pneumonitis is thought to arise from radiation-induced reactive oxygen species production, Deoxyribonucleic acid (DNA) damage, and disordered cytokine secretion. Excessive expression of cytokines such as transforming growth factor-beta 1 (TGF-β1) can promote pulmonary inflammation and subsequent fibrosis, which in severe cases necessitates radiotherapy interruption for supportive care. Myelosuppression results from radiation damage to hematopoietic stem cells, leading to reduced leukocyte counts. To avoid life-threatening infections, temporary treatment cessation is often clinically necessary to facilitate bone marrow recovery.

The observed associations between PS score 2–3, advanced disease, large tumor volume and treatment interruption reflect a high-risk patient profile shaped by multiple interacting clinical dimensions, although the present study design precludes any inference about causal pathways or mechanistic interactions. Patients with a PS score of 2–3 generally present with poor baseline physical status, limited treatment tolerance, and frequent comorbidities, all of which increase susceptibility to severe treatment-related adverse events and subsequent interruptions. Patients with advanced-stage lung cancer typically carry a high tumor burden, significant systemic consumption, impaired immune function, and possible distant metastasis. Disease instability and low treatment confidence further elevate the likelihood of interruption (32). When tumor volume is large, a larger radiation field and higher dose are usually required to achieve adequate local control, which intensifies normal tissue damage. In addition, bulky tumors may compress adjacent structures and aggravate clinical symptoms, further reducing patient tolerance and increasing interruption risk. Radiotherapy interruption adversely affects early clinical outcomes through several mechanisms. First, radiotherapy acts by delivering continuous high-energy radiation to disrupt tumor cell DNA and inhibit proliferation. Treatment breaks may allow cancer cells to repair sublethal damage and re-proliferate, weakening tumor control and reducing ORR and DCR (33). Second, prolonged overall treatment time facilitates accelerated tumor cell repopulation and raises the risk of disease progression. Third, radiotherapy interruption is usually triggered by severe toxicities, which themselves impair organ function, impede subsequent treatment, worsen immune dysregulation, and increase the risk of infectious complications, thus elevating the incidence of acute toxicities. Fourth, inferior tumor response, disease progression, and increased complications collectively contribute to compromised short-term survival, reflected by shortened PFS and OS. These observations highlight the clinical importance of maintaining uninterrupted radiotherapy to optimize early clinical outcomes (34).

A previous single-center retrospective study (35) reported that unplanned radiotherapy breaks reduced quality of life and overall survival, which is consistent with our observations. Another retrospective cohort study (36) identified treatment toxicity as the primary cause of interruption, similar to our findings. A multicenter prospective study (37) noted a 20% interruption rate among NSCLC patients undergoing radiotherapy, which is lower than the 35% observed in the present study. This discrepancy may be partially explained by differences in patient enrollment, that study included only NSCLC patients, whereas our cohort incorporated both NSCLC and SCLC. Variations in radiosensitivity and toxicity profiles across different pathological subtypes may also contribute to the divergent incidence rates.

The innovations of this study are multifaceted. First, we systematically characterized the temporal pattern of radiotherapy interruptions and verified that the majority occur during the middle one-third of treatment. These findings supplement the limited existing data on the phase distribution of treatment breaks and offer a time-directed basis for developing targeted clinical interventions. Second, we performed a detailed classification of interruption etiologies. Beyond confirming treatment-related toxicity as the dominant cause, we further specified the distribution of individual toxicities and patient-related factors. Compared with studies using relatively vague categorization, our results support more precise and individualized clinical management. Third, we comprehensively evaluated the impact of radiotherapy interruption on early oncologic outcomes, acute toxicities, and short-term survival, combined with risk factor analysis. In contrast to studies focusing on a single endpoint, our integrated approach provides a more complete understanding of the clinical implications of treatment interruption and offers a more robust evidence base for treatment optimization. In addition, the inclusion of tumor volume as a candidate risk factor helps refine the current risk profile for radiotherapy interruption in lung cancer, complementing existing literature in this field.

## 5 Limitations and future perspectives

Inevitably, the present work has several limitations that should be addressed and improved in future investigations. First, this study adopted a single-center retrospective design. All patients were recruited from the same institution, which means baseline characteristics, treatment protocols, and toxicity management strategies were relatively homogeneous. Such a design may introduce selection bias and restrict the generalizability of the findings, making it difficult to widely apply the conclusions to lung cancer patients treated in regions or hospitals with different clinical standards. Second, the follow-up period was limited to only 6 months, with focus solely on early clinical outcomes. The long-term effects of radiotherapy interruption on survival and late complications were not evaluated, so the full clinical significance of treatment breaks remains incompletely understood. Third, some data regarding patient-related factors were collected retrospectively, which carries a risk of information bias. Important variables such as age and comorbidities were not systematically documented, meaning potential confounders like immunosenescence and comorbidity control may have been overlooked. Fourth, this study did not analyze the graded effects of interruption duration and frequency on clinical outcomes. This was primarily due to the limited sample size of the interrupted group (n=79), which precluded robust subgroup analysis (e.g., stratification by 1-2, 3-5, >5 days) or reliable continuous modeling (such as restricted cubic spline analysis to identify potential threshold effects); consequently, we could not evaluate dose-response relationships between interruption severity and prognosis. Given that the events-per-variable rule would be violated with such a small sample, any attempt at refined duration-stratified analysis would yield unstable estimates and overly wide confidence intervals. As a result, we could not distinguish the harm associated with different severity of treatment breaks. In addition, the efficacy of salvage strategies after interruption was not explored, which may limit the precision of future intervention protocols. Although we adjusted for several key confounders (PS score, tumor stage, tumor volume, treatment regimen, and radiotherapy modality) in the multivariable Cox regression, we cannot rule out residual confounding from unmeasured factors, particularly age and comorbidity burden, which were not included due to sample size constraints (events-per-variable <10). The baseline comparability of these variables between groups (**Table 1**) does not fully eliminate the possibility of confounding, and future studies with larger samples are needed to address this issue. The present study could not reliably disentangle the independent contributions of radiotherapy interruption versus severe acute toxicity to survival outcomes. Constructing separate multivariable models with and without grade ≥3 toxicity would be subject to overadjustment bias, given that toxicity was the predominant cause of interruption and lies on the causal pathway. Moreover, the retrospective nature of our data precluded accurate temporal assignment of toxicity relative to interruption onset, and the limited sample size in the interrupted group (n=79) precluded meaningful stratified analyses. Future studies with larger sample sizes and time-to-event data are needed to address this question using time-dependent covariate models or mediation analyses.

Several inherent statistical limitations of our multivariable regression analysis should be acknowledged. First, the variable selection criterion (P<0.10 in univariate analysis) is an empirical convention commonly used in exploratory research, but it may introduce risks of overfitting and false-positive findings due to multiple testing. Second, our regression models were constructed for the purpose of assessing independent associations after adjusting for confounders, not for deriving a closed-form predictive equation. Hence, the adjusted effect estimates and P-values should be interpreted as exploratory evidence of association, not as confirmatory causal inference. Third, although the interrupted group (n=79) satisfied the rule of thumb for events-per-variable (~15.8 events per covariate), the sample size was still relatively modest, preventing us from applying more robust penalized regression methods (e.g., LASSO) or internal cross-validation to evaluate model generalizability. Consequently, these positive findings require external validation in independent, larger cohorts, and should be interpreted cautiously before being translated into definitive clinical decision-making.

For future research directions, multicenter and large-sample retrospective or prospective cohort studies are warranted. Enrolling patients from diverse geographic regions and clinical settings will improve population representativeness, reduce selection bias, and enhance external validity. An extended follow-up period is also recommended to systematically evaluate the impact of radiotherapy interruption on long-term survival, late toxicities, and quality of life. Further investigations should clarify the relationships between interruption duration, interruption frequency, and clinical endpoints, to quantify the risks associated with different interruption patterns and support individualized clinical interventions. It will also be valuable to incorporate immunological markers, comorbidity status, and other potential prognostic factors to refine risk prediction models. Interventional studies are needed to develop tailored supportive strategies for high-risk patients identified in this study. Combined with biomarker detection, a clinically applicable risk prediction model for radiotherapy interruption and severe toxicity can be established to enable early identification and precise management of vulnerable patients.

## 6 Conclusions

This study demonstrates that radiotherapy interruption occurs at a relatively high rate among patients with lung cancer, predominantly during the middle one-third of the treatment course. Treatment-related toxicity represents the most common cause of unplanned treatment breaks. Radiotherapy interruption may compromise treatment compliance and early oncological efficacy, increase the incidence of high-grade acute toxicities, and worsen short-term survival outcomes. Maintaining the continuity of radiotherapy appears to be critically important for optimizing early clinical outcomes in patients with lung cancer. These findings warrant further validation and refinement through future multicenter, prospective, and interventional clinical studies.

## Data Availability

The original contributions presented in the study are included in the article/supplementary material. Further inquiries can be directed to the corresponding author.

## References

[1] Nooreldeen R Bach H . Current and future development in lung cancer diagnosis. Int J Mol Sci. (2021) 22:8661. doi: 10.3390/ijms22168661 34445366 PMC8395394

[2] Smolarz B Lukasiewicz H Samulak D Piekarska E Kolacinski R Romanowicz H . Lung cancer-epidemiology, pathogenesis, treatment and molecular aspect (review of literature). Int J Mol Sci. (2025) 26:2049. doi: 10.3390/ijms26052049 40076671 PMC11900952

[3] Harethardottir H Jonsson S Gunnarsson O Hilmarsdottir B Asmundsson J Gudmundsdottir I . Advances in lung cancer diagnosis and treatment - a review. Laeknabladid. (2022) 108:17–29. doi: 10.17992/lbl.2022.01.671 34927601

[4] Deshpand R Chandra M Rauthan A . Evolving trends in lung cancer: epidemiology, diagnosis, and management. Indian J Cancer. (2022) 59:S90–105. doi: 10.4103/ijc.IJC_52_21 35343194

[5] Swaminath A Parpia S Wierzbicki M Kundapur V Faria S Okawara GS . Stereotactic vs hypofractionated radiotherapy for inoperable stage I non-small cell lung cancer: the LUSTRE phase 3 randomized clinical trial. JAMA Oncol. (2024) 10:1571–5. doi: 10.1001/jamaoncol.2024.3089 39298144 PMC11413752

[6] Spigel DR Faivre-Finn C Gray JE Vicente D Planchard D Paz-Ares L . Five-year survival outcomes from the PACIFIC trial: durvalumab after chemoradiotherapy in stage III non–small-cell lung cancer. J Clin Oncol. (2022) 40:1301–11. doi: 10.1200/JCO.21.01308 35108059 PMC9015199

[7] Desai P Edelman MJ Kumar S . State-of-the-art advances and management of unresectable locally advanced (stage III) non–small cell lung cancer. Am Soc Clin Oncol Educ Book. (2026) 46:e518252. doi: 10.1200/EDBK-26-518252 42234951

[8] Rodriguez Pla M Dualde Beltran D Ferrer Albiach E . Immune checkpoints inhibitors and SRS/SBRT synergy in metastatic non-small-cell lung cancer and melanoma: a systematic review. Int J Mol Sci. (2021) 22:11621. doi: 10.3390/ijms222111621 34769050 PMC8584181

[9] Mercier SL Moore SM Akurang D Tiberi D Wheatley-Price P . Stereotactic body radiotherapy (SBRT) in very limited-stage small cell lung cancer (VLS-SCLC). Curr Oncol. (2022) 30:100–9. doi: 10.3390/curroncol30010008 36661657 PMC9858162

[10] Tsai CJ Yang JT Shaverdian N Patel J Shepherd AF Eng J . Standard-of-care systemic therapy with or without stereotactic body radiotherapy in patients with oligoprogressive breast cancer or non-small-cell lung cancer (consolidative use of radiotherapy to block [CURB] oligoprogression): an open-label, randomised, controlled, phase 2 study. Lancet. (2024) 403:171–82. doi: 10.1016/S0140-6736(23)01857-3 38104577 PMC10880046

[11] Diez P Hanna GG Aitken KL van As N Carver A Colaco RJ . UK 2022 consensus on normal tissue dose-volume constraints for oligometastatic, primary lung and hepatocellular carcinoma stereotactic ablative radiotherapy. Clin Oncol (R Coll Radiol). (2022) 34:288–300. doi: 10.1016/j.clon.2022.02.010 35272913

[12] Zhao ZR Liu SL Zhou T Chen G Long H Su XD . Stereotactic body radiotherapy with sequential tislelizumab and chemotherapy as neoadjuvant therapy in patients with resectable non-small-cell lung cancer in China (SACTION01): a single-arm, single-centre, phase 2 trial. Lancet Respir Med. (2024) 12:988–96. doi: 10.1016/S2213-2600(24)00215-7 39305910

[13] Yan M Louie AV Kotecha R Ashfaq Ahmed M Zhang Z Guckenberger M . Stereotactic body radiotherapy for ultra-central lung tumors: a systematic review and meta-analysis and International Stereotactic Radiosurgery Society practice guidelines. Lung Cancer. (2023) 182:107281. doi: 10.1016/j.lungcan.2023.107281 37393758

[14] Heinzerling JH Mileham KF Robinson MM Symanowski JT Induru RR Brouse GM . Primary lung tumour stereotactic body radiotherapy followed by concurrent mediastinal chemoradiotherapy and adjuvant immunotherapy for locally advanced non-small-cell lung cancer: a multicentre, single-arm, phase 2 trial. Lancet Oncol. (2025) 26:85–97. doi: 10.1016/S1470-2045(24)00573-4 39615497

[15] Han BH Wang J . Chinese Medical Association guideline for clinical diagnosis and treatment of lung cancer (2024 edition). Chin J Oncol. (2024) 46:3175–213. doi: 10.3760/cma.j.cn112152-20240510-00189 39193605

[16] Yabe M Naito T Matsuda S Morita M Sekikawa M Miura K . Effect of cancer cachexia on the efficacy and treatment delivery of chemotherapy in older patients with advanced small cell lung cancer. Thorac Cancer. (2025) 16:e70104. doi: 10.1111/1759-7714.70104 40538154 PMC12179394

[17] Appel S Wilk L Saad A Urban D Sorotsky H Kalmanovitz G . Pneumonitis after chemoradiation followed by durvalumab for locally advanced lung cancer: predictors and effect on survival. Br J Radiol. (2025) 98:1436–45. doi: 10.1093/bjr/tqaf133 40577081

[18] Singh AK Dadey DY Rau MJ Fitzpatrick J Shah HK Saikia M . Blocking the functional domain of TIP1 by antibodies sensitizes cancer to radiation therapy. BioMed Pharmacother. (2023) 166:115341. doi: 10.1016/j.biopha.2023.115341 37625322 PMC12930424

[19] Daniello L Elshiaty M Bozorgmehr F Kuon J Kazdal D Schindler H . Therapeutic and prognostic implications of immune-related adverse events in advanced non-small-cell lung cancer. Front Oncol. (2021) 11:703893. doi: 10.3389/fonc.2021.703893 34268127 PMC8277237

[20] Daly ME Beagen P Madani MH . Nonsurgical therapy for early-stage lung cancer. Hematol Oncol Clin North Am. (2023) 37:499–512. doi: 10.1016/j.hoc.2023.02.002 37024386

[21] Al-Umairi R Tarique U Moineddin R Jimenez-Juan L Kha LC Cheung P . CT patterns and serial CT changes in lung cancer patients post stereotactic body radiotherapy (SBRT). Cancer Imaging. (2022) 22:51. doi: 10.1186/s40644-022-00491-1 36114585 PMC9482277

[22] McMillan MT Shepherd AF Kang M Lin L Shaverdian N Wu AJ . Safety and efficacy of stereotactic body proton therapy for high-risk lung tumors. J Radiosurg SBRT. (2023) 9:63–74. 38029007 PMC10681142

[23] Bai H Wang XF Xu YH Zaorsky NG Wang HH Niu GM . Brachial plexopathy following stereotactic body radiation therapy in apical lung Malignancies: a dosimetric pooled analysis of individual patient data. Radiother Oncol. (2024) 200:110529. doi: 10.1016/j.radonc.2024.110529 39255923

[24] Wong SK Hamm J Shokoohi A McGahan CE Ho C . Real world duration of curative intent breast, colorectal, non-small cell lung, and prostate cancer treatment. BMC Cancer. (2021) 21:215. doi: 10.1186/s12885-021-07923-4 33653306 PMC7923613

[25] Giuliani ME Filion E Faria S Kundapur V Toni Vu TTT Lok BH . Stereotactic radiation for ultra-central non-small cell lung cancer: a safety and efficacy trial (SUNSET). Int J Radiat Oncol Biol Phys. (2024) 120:669–77. doi: 10.1016/j.ijrobp.2024.03.050 38614279

[26] Li K Wang B Liu S Li C Cui H Qi Z . A retrospective analysis of the efficacy and safety of immune combination therapy in driver gene-negative non-small cell lung cancer with brain metastases. Cancer Treat Res Commun. (2025) 45:101015. doi: 10.1016/j.ctarc.2025.101015 41067130

[27] Song Z Li Y Chen S Ying S Xu S Huang J . Efficacy and safety of pyrotinib in advanced lung adenocarcinoma with HER2 mutations: a multicenter, single-arm, phase II trial. BMC Med. (2022) 20:42. doi: 10.1186/s12916-022-02245-z 35101045 PMC8805254

[28] Elaimy AL Schipper M Yin H Matuszak MM Dominello MM Bergsma DP . Prospective evaluation of non-small cell lung cancer radiation therapy treatment interruptions in a large statewide quality collaborative. Int J Radiat Oncol Biol Phys. (2024) 120:e19. doi: 10.1016/j.ijrobp.2024.07.1818 42574925

[29] Feng B Zheng Y Zhang J Tang M Na F . Chemoimmunotherapy combined with consolidative thoracic radiotherapy for extensive-stage small cell lung cancer: a systematic review and meta-analysis. Radiother Oncol. (2024) 190:110014. doi: 10.1016/j.radonc.2023.110014 37981084

[30] Xiao Y Wang Y Li J Cheng C Song C Wang X . Stereotactic body radiotherapy plus cadonilimab (PD-1/CTLA-4 bispecific antibody) as third-line or beyond therapy for refractory solid tumors: a phase 1b study. Cancer Commun (Lond). (2025) 45:1235–46. doi: 10.1002/cac2.70051 40693391 PMC12531412

[31] Yousafzai IK Mehreen A Noreen N Amanullah Ahmad H Fatima N . Frontiers in cancer and haematology: emerging biomarkers, therapeutics, and technologies. Curr Cancer Res. (2025) 1:54–68. doi: 10.64229/v6s2ft48

[32] Kuusisalo S Iivanainen S Koivunen JP . Association of anti-PD-(L)1 treatment duration to efficacy in advanced solid tumors: a single center retrospective study. Ann Med. (2025) 57:2476729. doi: 10.1080/07853890.2025.2476729 40091413 PMC11915729

[33] Xu Y Ren X Jiang T Lv S Gao K Liu Y . Circulating tumor cells (CTCs) and hTERT gene expression in CTCs for radiotherapy effect with lung cancer. BMC Cancer. (2023) 23:475. doi: 10.1186/s12885-023-10979-z 37226235 PMC10207832

[34] Yang L Wang C Zhang W Liu S Xuan T Jiang H . Iodine-125 brachytherapy treatment for newly diagnosed brain metastasis in non-small cell lung cancer: a biocentric analysis. Front Oncol. (2022) 12:1005876. doi: 10.3389/fonc.2022.1005876 36591479 PMC9797954

[35] Angiras RS Lobo D Senthiappan AM Banerjee S Challapalli S Sunny J . Impact of unplanned radiotherapy interruptions and prolonged overall treatment time on recurrence in head and neck squamous-cell carcinoma: a retrospective analysis from a single institution. Onco. (2026) 6:8. doi: 10.3390/onco6010008 30654563

[36] Moore AM Nooruddin Z Reveles KR Datta P Whitehead JM Franklin K . Durvalumab treatment patterns for patients with unresectable stage III non-small cell lung cancer in the Veterans Health Administration (VHA): a nationwide, real-world study. Curr Oncol. (2023) 30:8411–23. doi: 10.3390/curroncol30090611 37754526 PMC10529719

[37] Elaimy AL Higgins L Schipper M Yin H Matuszak MM Dominello MM . Treatment interruptions in NSCLC radiotherapy: impact on outcomes in a statewide quality collaborative. Int J Radiat Oncol Biol Phys. (2025) 123:e156. doi: 10.1016/j.ijrobp.2025.06.3637 42574925

